# Effect of an herbal extract Number Ten (NT) on body weight in rats

**DOI:** 10.1186/1749-8546-2-10

**Published:** 2007-09-14

**Authors:** David A York, Sonyja Thomas, Frank L Greenway, Zhijun Liu, Jennifer C Rood

**Affiliations:** 1 Pennington Biomedical Research Center, Louisiana State University System, Baton Rouge, Louisiana 70808, USA; 2 Louisiana State University Agricultural Center, Baton Rouge, Louisiana 70803, USA; 3 Center for Advanced Nutrition, Utah State University, 4715 Old Main Hill, Logan, Utah 84322, USA

## Abstract

**Background:**

Chinese herbal extract Number Ten (NT) is a dietary herbal formulation prepared from rhubarb, ginger, astragalus, red sage and tumeric. This study tested the effectiveness of NT in reducing body weight gain in rats.

**Methods:**

Sixty female Wistar rats were fed a high fat diet and acclimated to gavage feeding. The rats were divided into five treatment groups: (1) Control (n = 15); (2) NT-H (n = 15), 1.5 g/day; (3) NT-L (n = 10), 0.75 g/day; (4) Pr-fed (n = 10), pair fed to NT-H; (5) d-FF (n = 10), d-fenfluramine 2 mg/kg. Ten rats per group were sacrificed on day 56. Weight, food intake, clinical chemistry and body composition were evaluated. Five animals in the control and 1.5 g/day NT groups were left untreated during a two week recovery period.

**Results:**

The 0.75 g/day NT, 1.5 g/day NT, d-fenfluramine and pair fed groups gained 24.6%, 33.3%, 12.3% and 33.3% less than the control respectively (P < 0.0006). Leptin decreased 27.5% to 46.2% in the treatment groups vs. control (P < 0.009). Parametrial fat decreased 14.1% to 55.5% in the NT and pair fed groups vs. control (P < 0.006). The NT groups had soft stools, loss of hair around the mouth and coloration to the urine and stool without evidence of blood or bilirubin (attributed to chromogens in NT). There were no differences between groups in the clinical chemistry.

**Conclusion:**

This study demonstrated the efficacy of NT in reducing weight gain in rodents.

## Background

Herbal treatments have been found effective for major diseases such as cancer, diabetes mellitus, and cardiovascular disease [1-4]. We are looking for effective herbal treatments for obesity. Obesity has a major influence on the morbidity and life expectancy of people. Increasing incidence of obesity suggests this epidemic of overweight will only worsen in the future [5-7]. The absence of effective treatments for the grossly overweight, other than bariatric surgery, has encouraged the search for new drugs and drug targets that might be more effective in inducing weight loss and/or preventing weight gain [8,9].

Traditional Chinese herbal products were reported to be effective for the treatment of obesity [10,11]. One such, an herbal decoction known as Number Ten (NT, which is not a trade name) was reported in a published patent specification to be effective in preventing obesity in rats when it was orally gavage fed. NT is developed from *Rheum officinale *Baill. (*Dahuang*), *Astragalus complanatus *R. Br. (*Shayuanjili*), *Salvia miltiorrhiza *Bge. (*Danshen*), *Curcuma longa *L. (*Jianghuang*), and *Zingiber officinale *Rosc. (*Shengjiang*). The effect of NT to reduce body weight was reported in otherwise normal rats that had been made very obese by feeding a high energy diet [12] and also in obese rats that had been treated with monosodium glutamate (to induce hypothalamic lesions) [12]. In the former study, an improvement in blood lipid and blood glucose levels was also noted and in the latter one the obesity reducing effect was shown in rats of both genders. In a third study, NT was shown to be effective in attenuating the development of obesity without affecting the gain in lean body tissue in a young growing male rat that had been fed on high fat diet to induce obesity [12].

Animal models have been used extensively to test the efficacy of potential treatments for the prevention and treatment of obesity. In particular, rodents that are fed on high fat diet are thought to be an excellent model of obesity where dietary environment is a major contributor [13-15].

Using a similar model, namely rat on high fat diet, we report the efficacy of NT in preventing obesity and compare its actions to those of d-fenfluramine, a known anorectic [16,17].

## Methods

### Preparation of NT

The herbal decoction NT consists of a combination of 40% *Rheum officinale *Baill. (*Dahuang*), 13.3% *Astragalus complanatus *R. Br. (*Shayuanjili*), 13.3% *Salvia miltiorrhiza *Bge. (*Danshen*), 26–27% *Curcuma longa *L. (*Jianghuang*) and 6–7% *Zingiber officinale *Rosc. (*Shengjiang*). The rhubarb root and stem were placed in a stainless steel pot with water 6–8 times the herb's weight and a mixture of the other four herbs were prepared in a similar manner. The herbs were allowed to soak for eight hours. The water in the pot with the four-herb mixture was boiled until the volume was reduced by half. A cold rhubarb decoction was then added to the four-herb mixture and heated to just below boiling point for 20 minutes before cooling. After filtering the large particulates from the decoction, the remaining liquid was freeze-dried to a powder form, producing 0.5 g of solids from 10 ml of liquid.

### Animals

Sixty female Wistar rats (with an average weight of 220 g at the beginning of the study) were housed individually in hanging wire mesh cages. Room temperature was maintained at 22–24°C and light cycle was 12 hours with lights out at 18:00. The animals were accustomed to handling and daily gastric intubation over a one week period.

### Treatments

Rats were divided into five experimental groups. All were placed onto a high fat diet with 29% protein, 24% carbohydrate and 30% fat by weight for 56 or 70 days (energy density of diet = 4.78 kcal/g). Fresh food was placed in the cages immediately before lights out. The five groups of rats were tube-fed between 16:00 and 18:00 daily before lights out with water (Control, 15 animals), 1.5 g of freeze-dried NT powder (NT-H, 15 animals), 0.75 g of freeze-dried NT powder (NT-L, 10 animals), water while pair fed to NT-H (Pr-fed, 15 animals) and 2 mg/kg of d-fenfluramine (d-FF, 10 animals) respectively. The experimental protocol was approved by the Institutional Animal Care and Use Committee and conformed to all the federal guidelines.

### Food intake

Food intake was recorded daily throughout the experiment; body weight was recorded daily for the first 23 days and twice a week from day 23 to day 56. Except for 5 animals in the control and the 1.5 g NT groups, all rats were sacrificed by guillotine to collect trunk blood on day 56. The remaining ten animals were sacrificed on day 70. The parametrial and retroperitoneal fat pads were carefully dissected free of surrounding tissues by a single investigator to ensure consistency of dissection. Other tissues were individually dissected and weighed in a similar manner.

### Blood chemistry

Trunk blood was collected from fed rats between 10:00 and 14:00 and serum was prepared. Serum cholesterol, triglycerides, glucose, sodium, potassium, chloride, alkaline phosphatase, alanine aminotransferase, aspartate aminotransferase, creatine phosphokinase, gamma glutamyl transpepdidase, amylase were assayed on a Beckman Coulter Synchron CX7 chemistry analyzer using standard methods. Rat insulin, leptin and corticosterone were assayed by commercial radioimmunoassays (Linco Corp, San Diego, California, USA). Platelets, white blood cell count, mean cell volume, hematocrit and hemoglobin were also assayed on a Beckman Coulter HMX.

During the 56 days of the experiment, all rats were observed daily by an animal technician, and weekly by a veterinarian who was blind to the individual treatments of each rat. Notations were made regarding their physical activity/lethargy, fur condition, skin condition, eye condition, stools and 'others'.

### Body composition

Body composition of the eviscerated carcasses was measured. The frozen carcasses were allowed to warm to room temperature and then dried in a 60°C oven to constant weight. The dried carcasses were homogenized repeatedly to produce a homogeneous sample. A sample of this homogenate was used to assay protein (Kjeldahl analysis), fat (Soxhlet extraction) and ash (combustion) for calculation of body composition.

### Statistics

Data were analyzed using the Statistical Package for Social Sciences (SPSS) version 12. Weight, food consumption, body composition, organ weights and clinical blood analytes were analyzed using one-way analysis of variance (ANOVA) followed by a Scheffe Comparison of Means analysis for unbalanced groups. After sample sizes were balanced, an additional Bonferroni and Tukey Comparison of Means analysis was performed. Feed efficiency was calculated from the amount of weight gained in grams per gram of food consumed. Correlation was estimated by Pearson's coefficients. The results with P values less than 0.05 were considered to be statistically significant.

## Results

### Body weight

**Figure 1:**
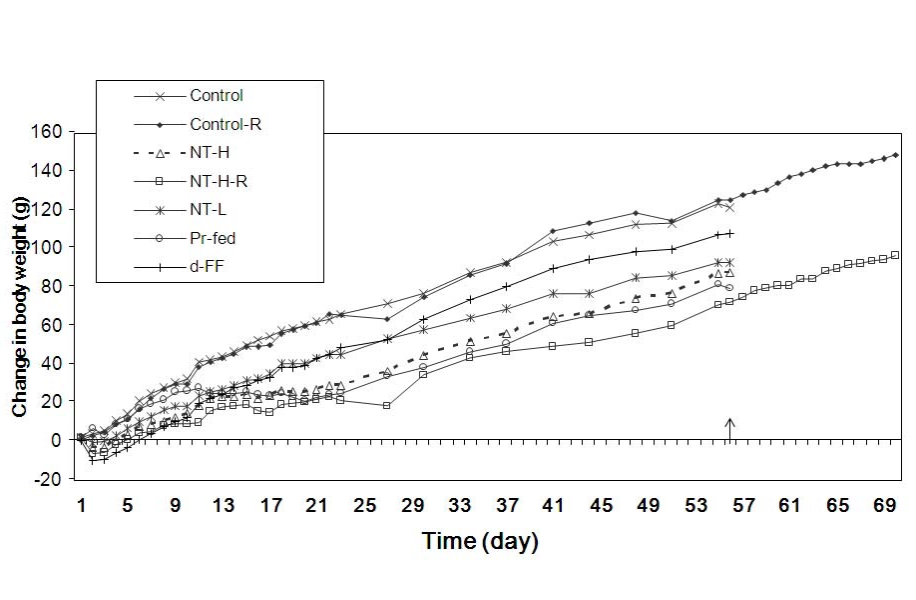
**Change in body weight during the treatment and recovery phases**. Values represent the means for each group. The arrow indicates completion of the treatment period and the start of the recovery period for the two groups that were not sacrificed at that point. NT-H: high dose of NT; NT-H-R: high dose NT with a two week recovery period; NT-L: low dose of NT; Pr-fed: pair fed to NT-H group; d-FF: d-fenfluramine.

**Figure 2:**
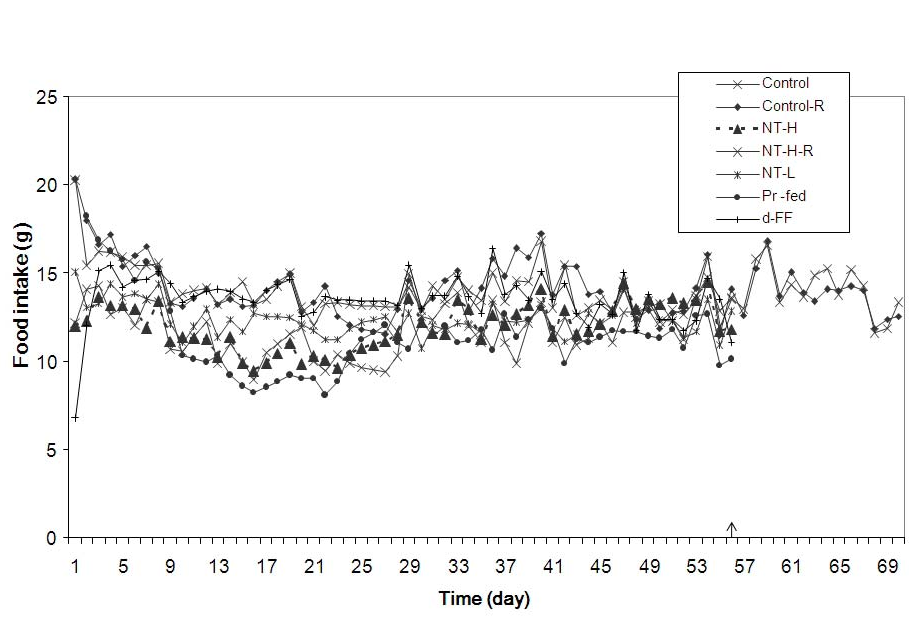
**Mean daily food intake**. The arrow indicates completion of the treatment period and the start of the recovery period for the two groups that were not sacrificed at that point. NT-H: high dose of NT; NT-H-R: high dose NT with a two week recovery period; NT-L: low dose of NT; Pr-fed: pair fed to NT-H group; d-FF: d-fenfluramine. The change in food intake of every group except the Control-R group was statistically different from the control group with P < 0.05 (d-FF and NT-L) or P < 0.01 (NT-H, Pr-fed and NT-H-R).

The mean body weights of the five experimental groups were similar at the start of the experiment. After the treatment of 56 days, the body weights of all experimental groups were significantly decreased compared to the control group (Figure 1). NT-treated groups and the d-fenfluramine (d-FF) treated group lost weight during the first 48 hours of treatment. After this time, the weight gain of the d-fenfluramine (d-FF) group paralleled that of the control group. By contrast, the NT-treated groups continued to gain weight at a slower rate than the control group. It should also be noted that the rats that were pair fed to the high dose NT group (NT-H) gained weight at the same rate as the NT-H group (Figure 2). On day 56 of the treatment, the rats administered low dose NT (NT-L) and high dose NT (NT-H) had gained 24.6% and 33.2% less weight than the control group respectively. The rats administered d-fenfluramine (d-FF) gained 12.3% less weight than the control group. After termination of treatment at 56 days, there was no increase in the rate of weight gain of the NT treated group (NT-H-R) over the next 14 days and their weight difference to the control group (Control-R) was maintained (Figure 1).

### Food intake

**Figure 3:**
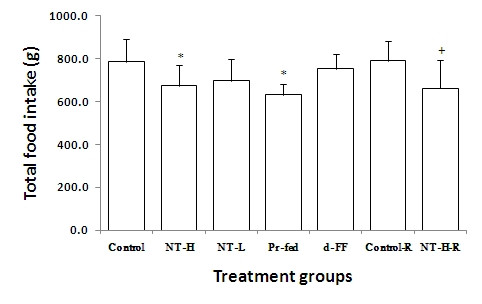
**Total food intake**. Values represent mean ± standard deviation (SD) for ten rats in each group except the two recovery groups (n = 5). Intake for the recovery groups is for 70 days whereas all other groups are for 56 days. * P < 0.05 compared to control group; + P < 0.05 compared to control recovery (Control-R) group. NT-H: high dose of NT; NT-H-R: high dose NT with a two week recovery period; NT-L: low dose of NT; Pr-fed: pair fed to NT-H group; d-FF: d-fenfluramine.

The food intake of each group over the experimental period is illustrated in Figures 2 and 3. The rats in the control group consumed significantly more total food than the rats in any of the treatment groups over the 56-day period. The rats administered d-fenfluramine (d-FF) consumed less total food over the 56 day period than the control group. On the other hand, they consumed significantly more food than those in other groups, namely NT-H, NT-L and Pr-fed. The pair-fed rats ate slightly less than the NT-H group during the experiment, presumably due to spillage of the diet.

### Feed Efficiency

**Figure 4:**
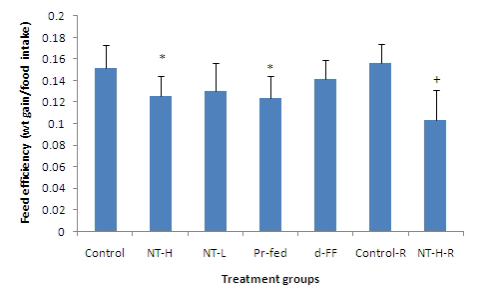
**Feed efficiency over the 56 day or 70 day (recovery groups) experimental periods**. Values represent mean ± SD for ten rats in each group except the two recovery groups (n = 5). * P < 0.05 compared to control group. + P < 0.05 compared to control recovery group. NT-H: high dose of NT; NT-H-R: high dose NT with a two week recovery period; NT-L: low dose of NT; Pr-fed: pair fed to NT-H group; d-FF: d-fenfluramine.

**Figure 5:**
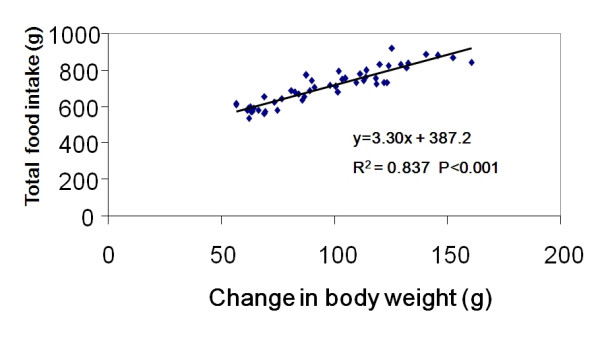
Correlation between food intake and body weight gain for all animals except those in the two recovery groups.

In this study, feed efficiency is expressed as weight gain (g) divided by weight of food consumed (Figure 4). The feed efficiency of the d-FF group, which was still higher than those of the NT-H, NT-L and Pr-fed groups, decreased significantly compared to the control group. The feed efficiencies of the NT-L and NT-H groups were 15.6% and 22.5% lower than that of the control group respectively. The feed efficiency of the d-FF group was 7.8% lower than that of the control group. Body weight gain and food intake were closely correlated in individual experimental groups (R^2 ^and P values for each group respectively: Control 0.719, P < 0.05; NT-H 0.907, P < 0.01; NT-L 0.796, P < 0.05; Pr-fed 0.694, P < 0.05; d-FF 0.798, P < 0.05) and in the combined data (Figure 5).

### Organ weights

There were no significant differences in the weights of the kidney, spleen, liver, and gastrocnemius muscle among the groups after the 56-day experimental period (data not shown). The hearts of the rats in the control group were significantly larger than those in all other groups. However, when the weight of the heart was taken as a percentage of the body weight of the respective rat, there were no significant differences between the groups.

**Figure 6:**
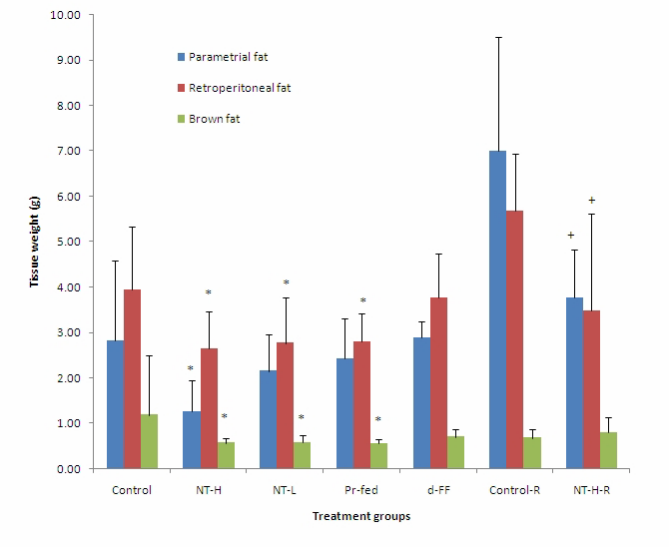
**Adipose tissue organ weights of rats**. Values represent mean ± SD for ten rats in each group except the two recovery groups (n = 5). * P < 0.05 compared to control group; + P < 0.05 compared to control recovery group. NT-H: high dose of NT; NT-H-R: high dose NT with a two week recovery period; NT-L: low dose of NT; Pr-fed: pair fed to NT-H group; d-FF: d-fenfluramine.

Parametrial and retroperitoneal white fat pads were significantly smaller in the NT-H group. They were also smaller in the NT-L and the Pr-fed groups but were not significantly different from either the NT-H group or the control group (Figure 6). There were no differences between the sizes of these adipose depots between the d-FF group and the control group. In the rats allowed to recover for two weeks after the end of treatment, the adipose depots remained smaller than those in the control group despite the fact that fat deposition increased significantly during the 2-week recovery period. The size of the inter-scapular brown adipose tissue depot was significantly reduced in all experimental groups compared to the control group (Figure 6).

### Body composition

**Figure 7:**
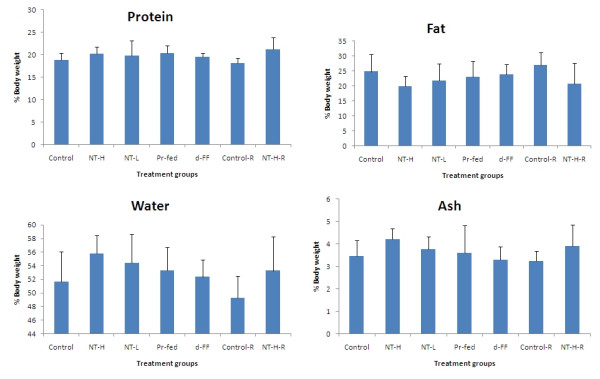
**Body composition**. Values represent mean ± SD for ten rats in each group except the two recovery groups (n = 5). NT-H: high dose of NT; NT-H-R: high dose NT with a two week recovery period; NT-L: low dose of NT; Pr-fed: pair fed to NT-H group; d-FF: d-fenfluramine.

**Figure 8:**
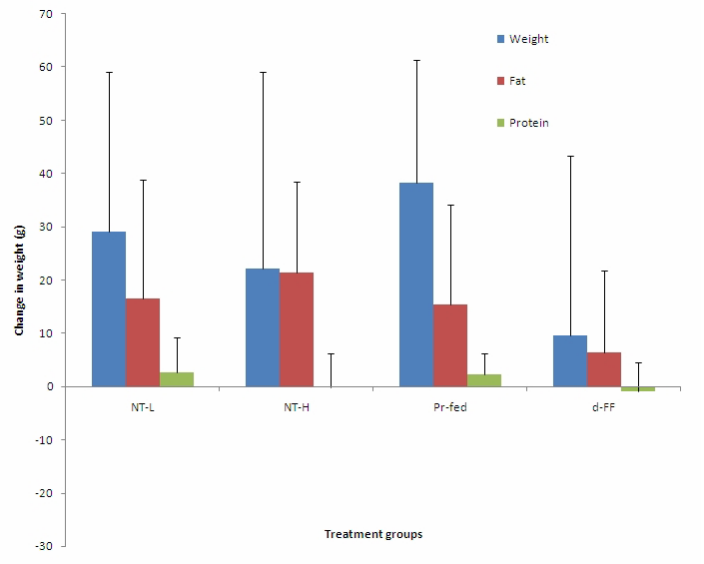
**Relative differences in body weight, body fat and body protein**. Values represent mean ± SD for ten rats in each group except the two recovery groups (n = 5). NT-H: high dose of NT; NT-H-R: high dose NT with a two week recovery period; NT-L: low dose of NT; Pr-fed: pair fed to NT-H group; d-FF: d-fenfluramine.

The percentage of total body fat was significantly reduced in the NT-H group and a clear dose-related reduction with NT treatment was observed (Figure 7), while body fat was not significantly altered in any other experimental groups. The percentage of body water, as expected, increased slightly in the NT groups. There were no significant effects of any treatment on body protein or ash (i.e. mineral content). Changes in body composition during the treatment period could not be calculated because no rats were sacrificed for analysis at time zero. However, from percentage compositions and final body weights, we calculated the differences in the body composition between the treatment groups and control group at the end of the experiment. Virtually all the differences in body weight between the NT-H group and control group can be attributed to the difference in body fat between these two groups (Figure 8).

### Serum hormones

**Figure 9:**
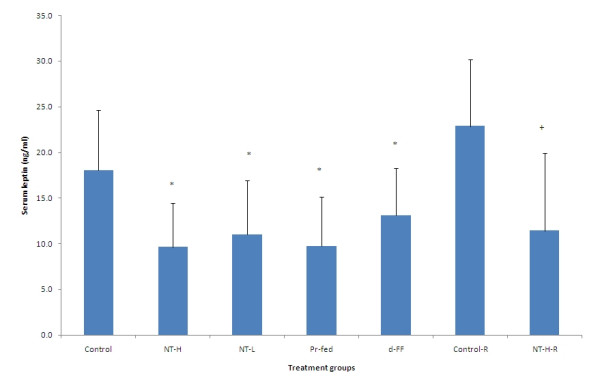
**Serum leptin levels**. Values represent mean ± SD for ten rats in each group except the two recovery groups (n = 5). * P < 0.05 compared to control group. + P < 0.05 compared to control recovery group. NT-H: high dose of NT; NT-H-R: high dose NT with a two week recovery period; NT-L: low dose of NT; Pr-fed: pair fed to NT-H group; d-FF: d-fenfluramine.

**Figure 10:**
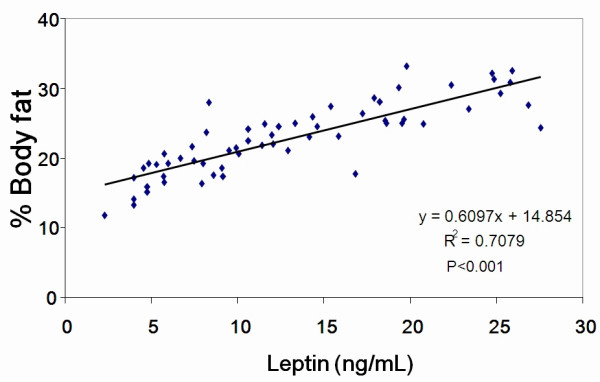
**Correlation between body fat and serum leptin**. Data include all animals except those in the two recovery groups.

Serum leptin was reduced significantly in all the treatment groups compared to the control group (Figure 9). The differences in leptin values continued during the 14-day recovery period despite the withdrawal of NT, which might be due to a modest weight gain during that period. Serum leptin levels were correlated with percentage body fat for each individual experimental group (R^2 ^and P values for each group: Control 0.887, P < 0.001; Control-R 0.627, P < 0.05; NT-H 0.855, P < 0.01; NT-H-R 0.99, P < 0.001; NT-L 0.933, P < 0.001; Pr-fed 0.875, P < 0.001; d-FF 0.769, P < 0.01) and the combined data (R^2 ^= 0.708, P < 0.001) (Figure 10).

### Serum metabolites

Serum prepared from trunk blood obtained at time of sacrifice (day 56) was used for clinical chemistry assays. The analyses included quantification of glucose, triglycerides, cholesterol, alkaline phosphatase, alanine aminotransferase, amylase, aspartate aminotransferase, chloride, cholesterol, creatinine phosphokinase, γ-glutamyl transpeptidase, glucose, hemoglobin, hematocrit, mean cell volume, platelets, potassium, sodium, triglycerides and white blood cells. One-way ANOVA plus Scheffes' Comparison of Means for each variable indicated that there were no significant differences between any of the serum analysis variables from the treatment groups and control group, nor among the treatment groups.

### Safety and toxicity

A small degree of alopecia during the first week of treatment was observed around the chins of the rats in the NT-H group. The alopecia disappeared within the next seven days after the animal technician was advised to be more scrupulous with the use of the intubation tube.

Rats in the NT treatment groups had yellowish urine which stained fur around the genitalia. Urine was tested negative for bilirubin. Rats in both NT-H and NT-L groups had loose stools for the first few days. By the end of the first week, the stools of the rats in the NT-L group were well formed and their consistency appeared normal, whereas the consistency of the stools of the rats in the NT-H group still appeared softer than those in other groups. Soft stools were observed among the rats in the NT-H group from time to time during the course of study. Moreover, the stools of the rats in both the NT-H and NT-L groups appeared reddish throughout the duration of the experiment. A test for hemoglobin was negative. The rats in the NT-H group liked to rub and groom the fur around their mouths after intubation, which was not observed in other groups. During the recovery period when the NT treatment was withdrawn, the color of the urine and stools returned to normal and the rats stopped grooming around the mouth. No other differences in activity/lethargy, fur condition, skin condition, eye condition or other physical characteristics were observed.

## Discussion

The results of this study demonstrate that NT was effective in reducing body weight gain in rats that had been fed a high fat diet to induce obesity. Rats in the high dose NT (NT-H) and low dose NT (NT-L) groups gained 33.2% and 24.6% less weight than those in the control group respectively. These results are consistent with the range of weight reductions in another study [12]. Furthermore, weight reduction by NT appeared to occur in a dose-dependent manner. Several interesting characteristics of the weight reduction are as follows.

Firstly, the temporal changes in weight induced by NT and d-FF were very different. Rats treated with d-FF showed the classical response of rodents to anorectic drugs whereby the majority of weight loss occurred in the first few days of treatment, after which weight gain paralleled that of the control group [16,18,19]. By contrast, the initial weight loss and reduction in food intake were less pronounced with NT treatment, instead a steady and continued reduction in weight gain throughout the study period was observed.

Secondly, rats in the pair fed group had a similar reduction in weight gain and body fat as the high dose NT group, therefore, reduction in weight gain with NT treatment appears to be caused by reduction in food intake rather than any metabolic effects. On the other hand, the failure of NT treated rats to regain body weight during the recovery period despite similar levels of food intake to the control group would conversely suggest the possibility of a small, prolonged metabolic effect. It also suggests that the NT treatment may lead to a long lasting reduction in body weight.

Thirdly, changes in body composition suggest that decreases in body weight can be attributed to decreases in body fat. Both the changes in the level of body fat and in the weight of the two fat pads are supportive of this interpretation. The close correlation of serum leptin levels to body fat provides great confidence in these data and once again confirms the observation that serum leptin levels are an excellent index of body fat levels [20]. There were no changes in body protein despite the reduction in food intake during this experimental period when the rats were still growing.

Overall, the data suggest that the effects of NT on body weight and body composition were mediated through changes in food intake rather than metabolic effects. The principal evidence for this is the similarity of the weight changes between the NT-H group and the Pr-fed group. It is important to recognize that the data on feed efficiencies should not be interpreted as indicative of any metabolic responses. Since the composition of the weight gain was different between control and NT-treated groups, the feed efficiency data underestimate the differences between control and NT-treated rats. However, the decrease in feed efficiency induced by NT reflects the differences in partitioning of energy into fat stores in the presence of reduced levels of food intake. The similarity in feed efficiency of NT-H group to the pair fed group support this suggestion. NT-treated rats protected their protein deposition at the expense of reducing their fat deposition, an entirely normal response in the face of diminished energy availability.

The mechanism through which NT reduced food intake is unknown. With any anorectic substances, it is important to ensure that the reduction in food intake is not induced through sickness or nausea. This is normally shown by performing a conditioned taste aversion assay (CTA) with the anorectic compound and comparing this to a known aversive agent such as lithium chloride [21]. A CTA test was not performed in the current study. There were no physical symptoms to suggest that the rats treated with NT were unwell or nauseated. On the contrary, the rats remained active, alert and in good physical condition throughout the study.

The rats treated with high dose NT that were allowed a two week post treatment recovery period did not exhibit any catch-up growth during this period. This finding may suggest a continued response to NT or storage of the active components within the body.

There were no clinical symptoms that would cause concern in using this product in humans. The change in appearance of stools was related to the dietary fat and chromogens in the herbs. The negative testing for blood and bilirubin along with the disappearance of these color changes in the recovery period are consistent with this hypothesis. The loss of hair around the mouth and nose was possibly a result of the rats trying to wipe off residual NT around the feeding cannula. This activity decreased when the external part of the cannula was cleaned of NT prior to intubation and ceased during the recovery period.

There is historical precedence for the use of rhubarb in the treatment of obesity in China [10] although this may be prohibited by regulatory bodies in some countries. While these animal studies are encouraging for the possible use of NT to treat human obesity, a recent clinical trial did not demonstrate any efficacy probably owing to the dose limiting gastrointestinal toxicity [22].

## Conclusion

NT is an effective herbal formulation for reducing weight gain in rats. Its effects are maintained throughout the treatment period as shown in the present study. No toxicity of NT was found in rats although gastric toxicity was reported in humans.

## Competing interests

The author(s) declare that they have no competing interests.

## Authors' contributions

DY designed the study, supervised the experiments and wrote the manuscript.

ST performed the animal experiments. FG provided advice on the NT supplement which was useful in the design of the study. ZL prepared the NT mixture and learned the procedure from Dr Kai-yuan Wei. JR supervised all the clinical chemistry assays in her laboratory. All authors read and approved the final manuscript.
